# Do Words Matter? Detecting Social Isolation and Loneliness in Older Adults Using Natural Language Processing

**DOI:** 10.3389/fpsyt.2021.728732

**Published:** 2021-11-16

**Authors:** Varsha D. Badal, Camille Nebeker, Kaoru Shinkawa, Yasunori Yamada, Kelly E. Rentscher, Ho-Cheol Kim, Ellen E. Lee

**Affiliations:** ^1^Department of Psychiatry, University of California San Diego, La Jolla, CA, United States; ^2^Sam and Rose Stein Institute for Research on Aging, University of California San Diego, La Jolla, CA, United States; ^3^Herbert Wertheim School of Public Health and Longevity Science, University of California San Diego, La Jolla, CA, United States; ^4^Digital Health, IBM Research-Tokyo, Tokyo, Japan; ^5^Cousins Center for Psychoneuroimmunology, Semel Institute for Neuroscience and Human Behavior, University of California, Los Angeles, Los Angeles, CA, United States; ^6^AI and Cognitive Software, IBM Research-Almaden, San Jose, CA, United States; ^7^VA San Diego Healthcare System, La Jolla, CA, United States

**Keywords:** artificial intelligence, social connectedness, gender, loneliness, NLP, Social support, linguistic features

## Abstract

**Introduction:** Social isolation and loneliness (SI/L) are growing problems with serious health implications for older adults, especially in light of the COVID-19 pandemic. We examined transcripts from semi-structured interviews with 97 older adults (mean age 83 years) to identify linguistic features of SI/L.

**Methods:** Natural Language Processing (NLP) methods were used to identify relevant interview segments (responses to specific questions), extract the type and number of social contacts and linguistic features such as sentiment, parts-of-speech, and syntactic complexity. We examined: (1) associations of NLP-derived assessments of social relationships and linguistic features with validated self-report assessments of social support and loneliness; and (2) important linguistic features for detecting individuals with higher level of SI/L by using machine learning (ML) models.

**Results:** NLP-derived assessments of social relationships were associated with self-reported assessments of social support and loneliness, though these associations were stronger in women than in men. Usage of first-person plural pronouns was negatively associated with loneliness in women and positively associated with emotional support in men. ML analysis using leave-one-out methodology showed good performance (F1 = 0.73, AUC = 0.75, specificity = 0.76, and sensitivity = 0.69) of the binary classification models in detecting individuals with higher level of SI/L. Comparable performance were also observed when classifying social and emotional support measures. Using ML models, we identified several linguistic features (including use of first-person plural pronouns, sentiment, sentence complexity, and sentence similarity) that most strongly predicted scores on scales for loneliness and social support.

**Discussion:** Linguistic data can provide unique insights into SI/L among older adults beyond scale-based assessments, though there are consistent gender differences. Future research studies that incorporate diverse linguistic features as well as other behavioral data-streams may be better able to capture the complexity of social functioning in older adults and identification of target subpopulations for future interventions. Given the novelty, use of NLP should include prospective consideration of bias, fairness, accountability, and related ethical and social implications.

## Introduction

“*No man is an island entire of itself; every man is a piece of the continent, a part of the main…”*—John Donne.

Rates of social isolation and loneliness (SI/L) have increased over the past few decades among older adults, impacting both mental and physical health (1, 2). SI/L is associated with increased alcohol and drug abuse (3), cognitive decline (4), development of depressive and anxiety symptoms (5, 6), poor physical functioning (7–9), as well as increased mortality (10, 11). Furthermore, the adoption of physical distancing guidelines during the COVID-19 pandemic has further isolated seniors from relationships and meaningful activities, impacting health and well-being (12).

While current studies rely on self-report measures of social interactions and subjective experiences to assess SI/L, these approaches may not fully capture the nature or quality of the social connections. Our previous work has used Natural Language Processing (NLP) approaches to identify subtle speech-based linguistic features that reflect loneliness in older adults. We found strong gender differences in the acknowledgment of loneliness and expressed sentiment among older adults (13). These findings provided foundational support that unstructured text data can provide unique insights into internal subjective experiences, including for the detection and understanding of SI/L. Building upon our previous work, the current study examined how older men and women describe relationships and social supports during a semi-structured interview. This NLP analysis was applied to interview segments that focused on social relationships (where loneliness was not specifically mentioned), successful aging, and technology.

We hypothesize that linguistic features may be reflective of SI/L due to the social nature of language, which often reflects how an individual relates to others. Linguistic data may provide a novel data source for understanding and assessing SI/L and may be particularly useful to social media companies, as such data is widely accessible unlike clinical and psychological measures including depression. For example, an individual who is lonely may have higher usage of first-person singular pronouns (“I”) than that of first-person plural pronouns (“we”). This may reflect a lack of social contacts, close family members, or significant others, as well as signal a lack of closeness or commonality with social contacts. Similarly, an individual who is lonely may feel distressed over a lack of social relationships and would use more negative language to describe them to an interviewer. In addition to pronoun usage, we also explored use of other parts-of-speech and syntactic complexity in relationship to SI/L. While few NLP studies have explored this previously, there have been links between socioeconomic status and education with loneliness—which could be indirectly reflected by syntactic complexity (longer and more complex sentence structures) or diversity of language used.

In this proof-of-concept study, we explored the interplay of gender and SI/L on transcribed speech data, using validated self-report scales for SI/L and NLP techniques, to provide a qualitative assessment of relationships. We examined the association between a scale-based measure of social support with the number and type of described relationships. We also examined how textual features, in particular pronoun usage, reflected self-report ratings of SI/L. Last we created machine learning (ML) models to predict SI/L based on sociodemographic and linguistic -based features, comparing the top-ranking features for different aspects of social support and loneliness.

## Research Design and Methods

### Participants and Procedures

For this study, we utilized data collected *via* interviews with residents living independently at a continuing care senior housing community (CCSHC) in southern California. Cohort characteristics and study procedures have been previously published (14, 15). The study was approved by the University of California San Diego Human Research Protections Program (HRPP). Study inclusion criteria were: (1) English speaking individuals 65+ years old, (2) Ability to complete study assessments and engage in a qualitative interview, and (3) No known diagnosis of dementia or any other disabling illness.

### Sociodemographic and Clinical Measures

Sociodemographic data including age, gender, racial background, years of education and marital status were collected along with scales to measure depression (Patient Health Questionnaire, 9-item) (16) and anxiety (Brief Symptom Inventory—Anxiety subscale).

### Measures of Social Functioning

Social support was assessed using scales from the MacArthur Studies of Successful Aging that included measures of Emotional Support (6-item scale, including “How often does your spouse make you feel loved and cared for?”), Instrumental Support (6-item scale, including “How often does your spouse help with daily tasks like shopping, giving you a ride, or helping with household tasks?”) and Negative aspects of Social Relationships (6-item scale, including “How often does your spouse make too many demands on you?”) (17).

Loneliness was assessed with the UCLA Loneliness scale (Version 3) or UCLA-3, a validated and commonly used research instrument. The UCLA-3 has high internal consistency, validity, and test-retest reliability (18). Unlike single-item assessments of loneliness, the UCLA-3 does not explicitly use the word “lonely.” The 20 items inquire about specific experiences, e.g., “How often do you feel in tune with others around you?” using a 4-point Likert scale (1 = “I never feel this way” to 4 = “I often feel this way”). The cut-offs for loneliness severity on the UCLA-3 scale were adapted from Doryab et al. (19), such that total scores ≤ 40 are categorized as not lonely and total scores >40 are categorized as lonely. Q2 (What makes those meaningful to you?) was included in extraction of linguistic features. However, due to the open-ended scope of the question and lack of concrete or objective information for further analyses, we only included linguistic features from those responses. Some of the commonly used social scales are henceforth referred to using acronyms: ESS-E, Emotional Support Scale—Emotional Support score; ESS-I, Emotional Support Scale—Instrumental Support; ESS-NI, Emotional Support Scale—Negative Interaction Score; SSI, Social Support Index.

### Qualitative Interviews

In addition to the aforementioned data collection, semi-structured interviews were conducted with participants covering a variety of topics (loneliness, relationships, and wisdom). Interviews were conducted by research staff trained in qualitative methods (Patton 2002) and occurred between April 2018 and August 2019. The interview protocol included six questions on the topic of relationships: (Q1) “So, this first section is about family, friendships and relationships. Do you have important relationships in your life? Please describe them.” (Q2) “What makes those relationships meaningful to you?” (Q3) “Do you feel that there are people in your life who fully understand you?” (Q4) “How often do you spend time with or connect (*via* phone, email, or social media) with others?” (Q5) “Do you feel you are part of a larger community? Please explain.” (Q6) “When you are feeling disconnected or isolated what do you do?” Each interview was audio-taped and subsequently transcribed by a commercial company (MModal). The interviews were manually transcribed verbatim and distinguished between the interviewer and interviewee. The same interviewer conducted all the interviews. This study focused upon Q1 responses to extract the number of important relationships, Q3 responses to extract the number of relationships in which one felt understood, and Q4 responses to extract frequency and mode of communication. The relationship section of the interview was used to extract linguistic features since these questions were consistent between the self-reported lonely and not-lonely, whereas for pronoun usage, we used the entire interview text in addition to the relationship section, given that focusing the conversation upon relationships could bias the pronoun usage (e.g., increased use overall of pronouns to describe their social network).

### Analytic Procedures

NLP techniques allow us to isolate relevant pieces of information within a response and suitably encode the information into numerical values or “features.” Some of these features are derived from the entire transcript, while others are derived from responses to specific questions or an entire thematic section. Many of these features are present in varying strengths, commonly referred to as “impurity” levels in NLP analysis, among classes based on user-defined criteria (e.g., gender, loneliness levels). This impurity of features (probability of incorrectly classifying) is exploited by ML techniques to discriminate among the classes even if the impurity is not significant, or the association is non-linear, or if several features must be composed together for the ML analyses. The following subsections discuss the steps involved and implementation details.

### Text Processing to Localize Responses

Term Frequency—Inverse Document Frequency (TF-IDF) techniques (20, 21) were used to identify specific questions and subsequent responses. These TF-IDF techniques are commonly used in document retrieval and data mining approaches (22). Briefly, within this method, the transcript of the interview is akin to a “corpus,” the entirety of text to be searched. Each question in the actual interview is analogous to a “document,” which must be matched (and its location retrieved) to a template question of interest, or a “query.”

Matching the query with the document uses vector algebra. First, the corpus (or collection of documents) is converted into vectors to capture the frequency (TF component) and the uniqueness (IDF component) of words (henceforth referred to as “terms”). Next, the queries are also vectorized. Finally, the query vectors can be matched with document vectors (using cosine-similarity) to identify best matches. The procedure is repeated for each transcript.

The transcribed interviews identified the interviewer's utterances with a new line preceded by the character “Q,” while the interviewee's answers were preceded by the character “A.” TF-IDF implementation queries were used with the actual questions in the transcripts. The TF-IDF approaches allowed text to be identified within each transcript that best matched the template query. After identifying the location of the question, we extracted the subsequent response (several lines following the “A” in the transcribed interview text).

### Text Processing to Extract Information

#### Linguistic Features

Linguistic features include frequency and ratio of parts of speech, vocabulary richness (Brunét's index, Honore's statistic, type token ratio), filled pauses (dysfluency in speech), syntactic complexity (complex and compound phrase structure within a sentence), sentence similarity (similarity between all pairs of sentences), and sentiment (23). For sentiment analysis, we used VADER (Valence Aware Dictionary for Sentiment Reasoning) a highly regarded and freely available tool. VADER is sensitive to polarity (positive/negative) as well as the strength of conveyed emotions. VADER is based on a dictionary which maps words into sentiment values (covering the positive to negative range), and also rates text based on capitalization and punctuation. VADER is ranked as one of the best in a 2016 benchmark study of commonly used sentiment analyzers (24). Once the location of relevant text in the transcripts was identified, a variety of techniques were used to quantify the represented information. As previously mentioned, all linguistic features, aside from pronoun usage, were extracted from only the relationship section of the interview. Specific details on these features are available in the Supplementary Appendix A.

#### Pronoun Usage-Based Features

We computed the density of first-person singular (I, me, my, and mine), first-person plural (we, our, us, and ours) and the third-person plural (he, she, they, them, and their) pronouns, but excluded the second-person pronouns (you, your, and yours) because they were primarily used to address the interviewer in the transcripts. Although these features are also linguistic in nature, they are mentioned in a separate category due to the nuanced semantics conveyed about relationships with others. Due to the focus on relationships with others in the relationship section, the section had higher pronoun usage and effect sizes were small to very small (<0.20). We used the transcript from the entire interview for pronoun-related analyses, which provided higher discrimination.

#### Relationship Word-Based Features

A dictionary of words was manually created to identify relationships mentioned by participants in their responses. These relationship words were further mapped into categories, e.g., “husband” and “wife” were categorized into “spouse.” Supplementary Table 1 outlines the mapping between relationship words and assigned categories. We also created a dictionary of predefined phrases that are often used in American English to identify modes and frequency of communication. To assess communication frequency, the phrases were mapped to approximate frequency as shown in Supplementary Table 2.

### ML Classification

**Figure 1:**
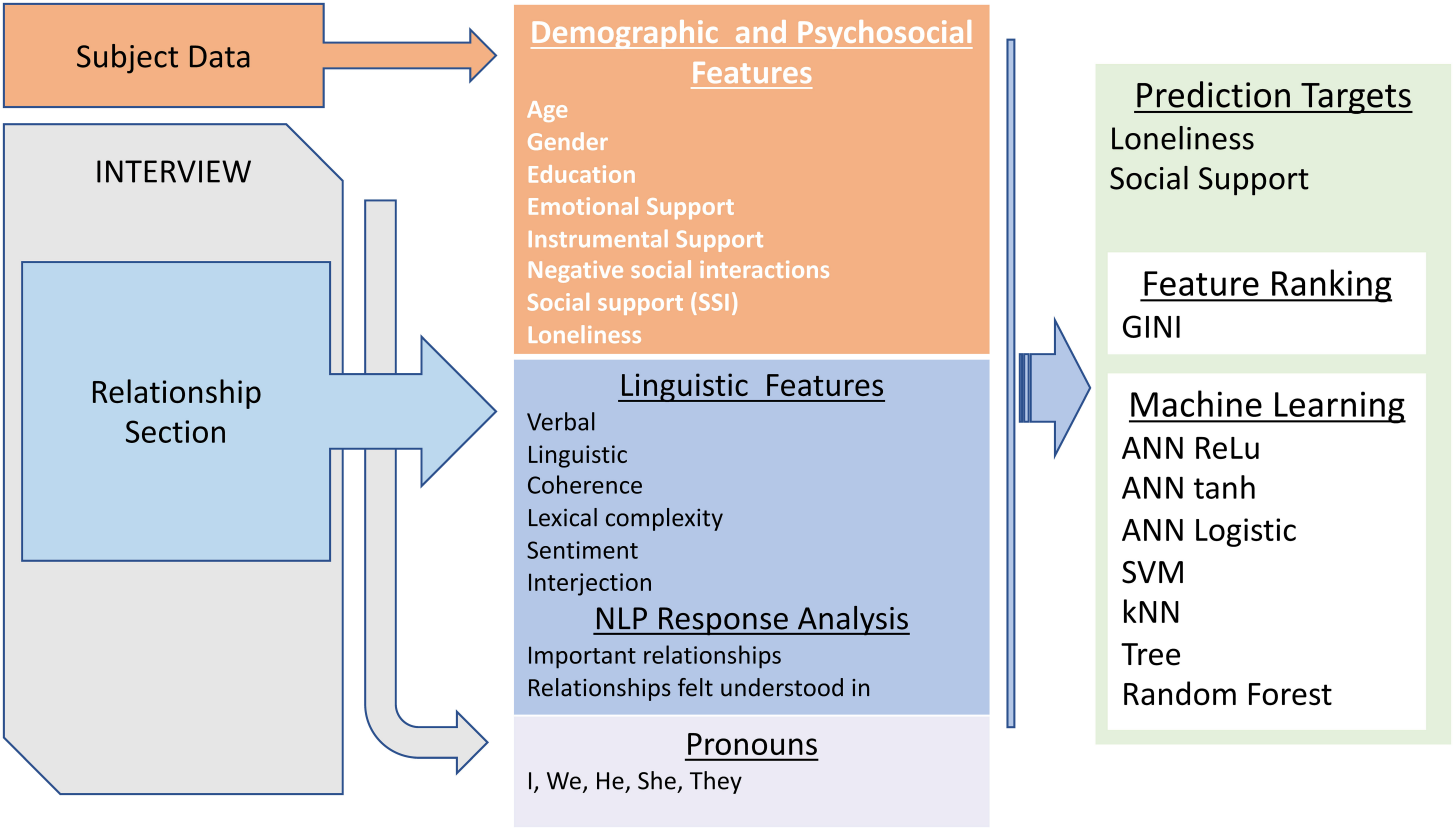
Overview of data analysis.

Socio-demographic features (education, age, race, marital status, etc.), linguistic features, and all pronoun density features (*N* = 97) were used to classify participants into objective categories for loneliness (UCLA-3 severity, cutoff score of 40) and social support (median cutoff) using Artificial neural network (ANN) with 200 internal units in Orange version 3.27.1, scikit-learn version 0.24.2. (25) was used. Various ML models such as Artificial neural network (ANN) with activation functions (Logistic, ReLu, and tanh), support vector machine (SVM), k-nearest neighbors (kNN), Tree and random forest were used (25). Figure 1 depicts the overall procedure along with features and sources used for our processing. Performances of binary-classification models were evaluated by using F1 score and the area under receiver operating characteristic curve (AUC) with leave-one-subject-out cross validation.

### Feature Ranking

Classifiers usually benefit from a large feature set, however, as the size of the feature set grows, at some point, error rates begin to increase (26). This phenomenon becomes even more relevant as the size of the feature set becomes comparable to the sample size, as in our case. There is a strong possibility of overfitting, and many features may be a source of noise. The approach usually (27, 28) is to rank features and then use top features incrementally to find the best performing set. This usually results in improved performance.

To determine the top-ranking NLP features that contribute to SI/L, we assessed how differently the feature is distributed across classes (previously referred to as impurity). GINI is a popular impurity-based feature ranking technique (29) that states the probability that the feature is wrongly classified (0 = “pure,” 0.5 = equal distribution across all classes, 1 = random distribution across classes) (29, 30). GINI was used to rank the features that were most strongly associated with the SI/L classification.

### A Caveat on Anaphora and Overestimation

Using NLP to extract information about relationships from unstructured text has a few notable challenges. For example, a response may mention “I have children. A son and a daughter.” Such responses require establishing correspondences between nouns (and pronouns), possibly separated by long spans of text. Anaphoric resolution (establishing correspondence among nouns and pronouns that refer to the same entity within and across sentences) is difficult, hence we acknowledge the possibility of overestimation in this process (31, 32). Our analysis relies upon counting words from our dictionary of relationship terms. Thus, our NLP-guided count of relationships may overestimate the intended number of relationships in the response due to possible anaphoric references.

## Results

Of the 101 interviews, 97 participants also completed other baseline assessments and were included in the analyses for this study.

### Description of the Study Sample

Participants ranged between 66 and 94 years of age (Table 1). Men were older (Mean age = 86.2 vs. 81.7 years for women, Cohen's *d* = −0.68) and had more years of education (Cohen's *d* = −0.40) than women. Racial background, marital status, mean UCLA-3 scores, instrumental support, negative interactions, anxiety, and depression scores were similar by gender.

**Table 1:** Demographics information.

	**Women**				**Men**							
	N*	**MEAN**	**MEDIAN**	**SD**	N*	**MEAN**	**MEDIAN**	**SD**	**t or χ^2^**	**df**	**p**	**Cohen's d**
Age at visit (years)	63	81.7	81.5	6.94	34	86.2	86.5	5.90	−3.36	96	<0.001	−0.68
Education (years)	63	15.4	16.0	2.42	34	16.4	16.0	2.23	−1.95	96	0.06	−0.40
Race (% Caucasian)	63	90.5			34	94.1			0.06	1	0.81	
Marital Status (% not single)	63	34.9			34	52.9			2.26	1	0.13	
Loneliness (UCLA-3 score)	54	36.2	35.0	9.35	30	39.3	38.5	11.54	−1.24	83	0.22	−0.30
Emotional support (ESS-E)	60	2.8	3.0	0.41	33	2.6	2.5	0.47	1.97	92	0.05	0.44
Instrumental support (ESS-I)	60	2.0	2.0	0.85	33	1.9	2.0	0.79	0.59	92	0.55	0.13
Negative social interactions (ESS-NI)	60	0.7	0.5	0.72	33	0.8	0.5	0.65	−0.98	92	0.33	−0.21
Social support (SSI)	53	52.0	52.0	7.41	31	49.6	50.0	7.24	1.41	83	0.16	0.32
PHQ-9	57	2.7	2.0	3.55	31	3.5	2.0	3.89	−0.91	87	0.37	−0.21

* *N refers to number of available observations at baseline. Some information was incomplete (unavailable)*.

*ESS-E, Emotional Support Scale—Emotional Support score; ESS-I, Emotional Support Scale—Instrumental Support; ESS-NI, Emotional Support Scale—Negative Interaction Score; PHQ-9, Patient Health Questionnaire 9-item; SSI, Social Support Index; UCLA-3, UCLA Loneliness Scale (Version 3)*.

### Comparison of Self-Report and NLP-Based Measures of Social Support

**Figure 2:**
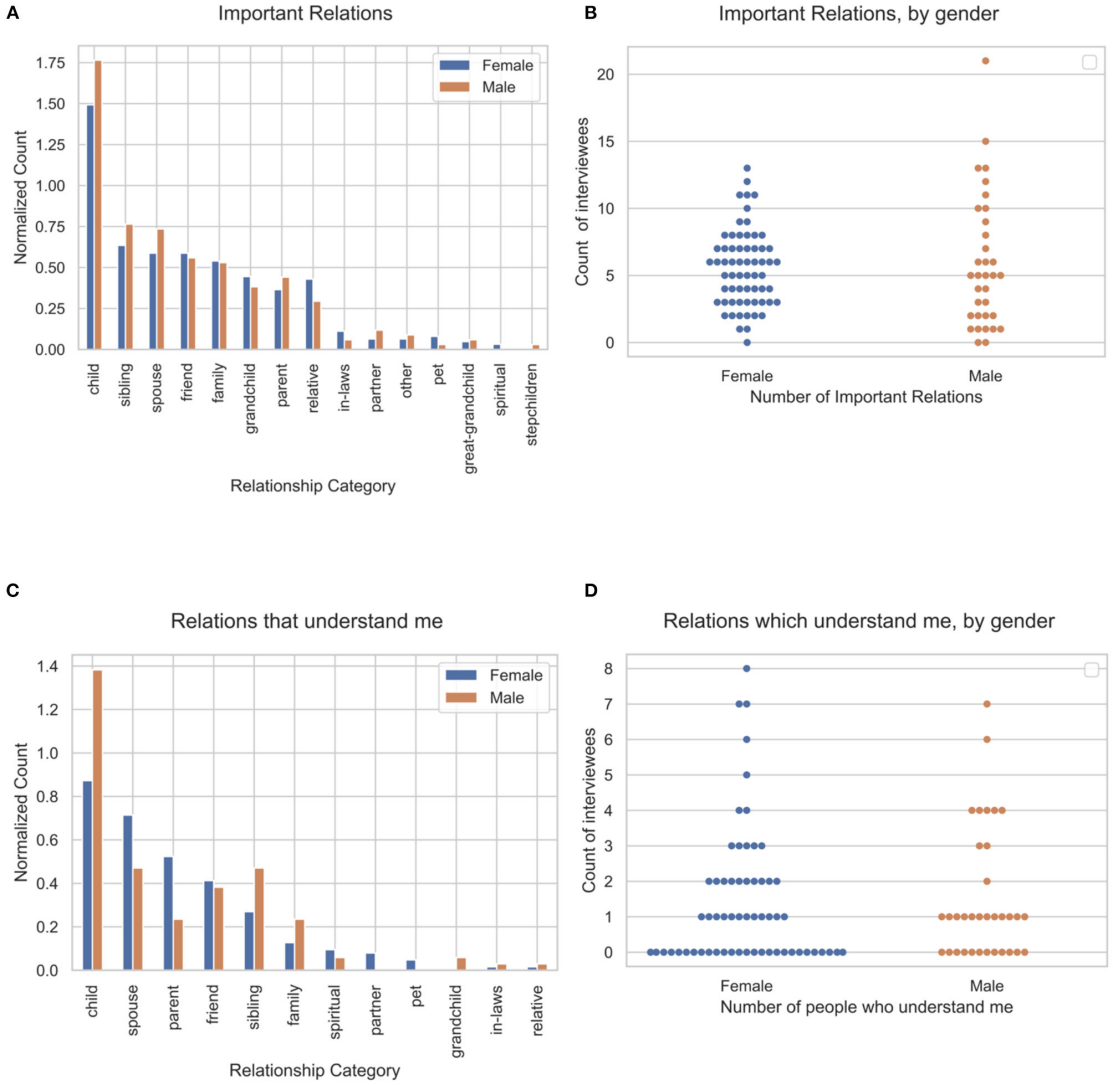
Number and type of important relationships and relationships in which one feels understood. **(A)** Relationship category, **(B)** no significant difference in the distribution of the counts by gender, **(C)** relations that understand in response to Q3, and **(D)** no significant difference in the distribution of the counts by gender.

The location of responses corresponding to Q1 and Q3 in the transcripts were identified correctly for all 97 interviewees, and more than 97% of responses were captured for the analyses. Figures 2A,B show the relationship type and distribution of important relationship terms by gender, in response to Q1. The identified relations were mapped into relationship categories. Children were most commonly reported as important relationships (63.5% women, average 1.5 mentions per interviewee for women overall, 52.9% men averaged 1.76 mentions per interviewee overall for men), followed by siblings and spouses. Figures 2C,D show, by gender, the relationship type and distribution of relationships in which the participant feels understood. In terms of feeling understood, participants most commonly noted children, spouses, and parents. Women and men reported similar numbers of important relationships (Mann–Whitney *U* = 993.0, *p* = 0.18, Cohen's *d* = −0.084) and relationships in which they felt understood (Mann–Whitney *U* = 989.5, *p* = 0.26, Cohen's *d* = −0.085). A sizable fraction of men (35.2%) and women (46.0%) reported they were not understood by anyone.

**Figure 3:**
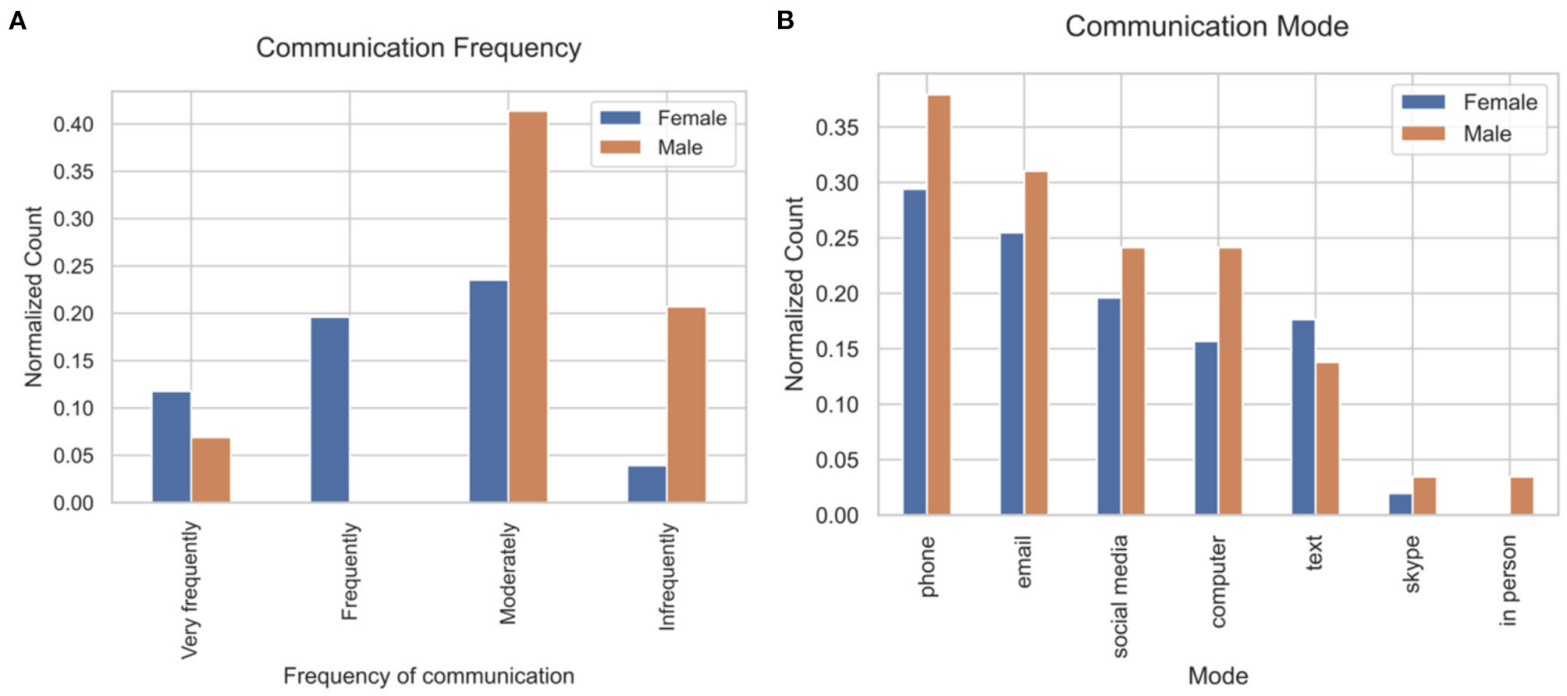
Frequency and mode of communication with social network. **(A)** Communication frequency and **(B)** communication mode. Details are shown in Supplementary Table 2.

Women communicated with their social network more frequently than men based upon key phrases in response to Q4 mapped to frequency (23.5 times a month vs. 8.0 times a month, Mann–Whitney *U* = 131.5 *p* < 0.001, Cohen's *d* = 0.76; Figure 3A). The most frequently mentioned mode of communication was phone (*N* = 26), followed by email (*N* = 22), and social media (*N* = 17), which included Facebook and Instagram (Figure 3B).

Emotional and instrumental support were associated with the NLP-derived assessments of relationships. The number of important relationships was correlated with greater emotional support in women, but not men (Spearman's ρ = 0.28, *p* = 0.03 and Spearman's ρ = −0.06, *p* = 0.73, respectively). Furthermore, the number of important relationships was negatively correlated with negative social interactions in women, but not in men (Spearman's ρ = −0.34, *p* = 0.009 and Spearman's ρ = 0.11, *p* = 0.55, respectively). The numbers of important relationships were not significantly correlated with UCLA-3 loneliness scale scores (Spearman's ρ = −0.15, *p* = 0.16) in either gender.

### Text Features Related to SI/L: Pronoun Usage

The density of types of pronouns, computed as a ratio of their occurrence counts divided by total number of words uncovered several interesting associations. First-person plural pronoun usage negatively correlated with loneliness in women (ρ = −0.31, *p* = 0.025). Emotional support in women was directly related to third-person pronoun density (Spearman's ρ = 0.30, *p* = 0.008).

### Binary Classification Models and GINI Based Feature Ranking

The ANN using Logistic activation function outperformed the others (F1 = 0.73, AUC = 0.75, specificity = 0.76, and sensitivity = 0.69) in predicting loneliness. This approach also performed similar to our previous approach for quantitative loneliness (13). Performance for ML for various measures such as loneliness and social support are shown in Tables 2, 3. Social support classification using ML showed acceptable performance (ESSES: F1 score = 0.67, AUC = 0.72; ESSIS: F1 score = 0.66, AUC = 0.71; ESSNS: F1 score = 0.67, AUC = 0.62) for median split.

**Table 2:** Binary-classification performance with loneliness (Leave one out)^*^$.

				**Confusion matrix**			
**Models**	**Hyper parameters**	**AUC**	**F1 Score**	**TP**	**FP**	**TN**	**FN**
ANN Logistic	Scipy implementation, Number of hidden layers = 1, Number of neurons in hidden layer = 200, solver = Adam	0.75	0.73	24	12	37	11
SVM	Cost (C) = 1.00, Numerical Tolerance = 0.001, Epsilon = 0.10, g = auto, kernel = RBF	0.74	0.73	23	11	38	12
ANN tanh	Scipy implementation, Number of hidden layers = 1, Number of neurons in hidden layer = 200, solver = Adam	0.67	0.65	20	14	35	15
ANN ReLu	Scipy implementation, Number of hidden layers = 1, Number of neurons in hidden layer = 100, solver = Adam	0.70	0.64	21	16	33	14
Tree	Max depth = 100, Min number of instance in leaves = 1	0.59	0.57	19	20	29	16
Random Forest	Number of Trees = 8, Number of attributes for split = 4, Limit depth = 7, Don't split subsets smaller than 2	0.57	0.55	12	14	35	23
kNN	Number of neighbors k = 9, Metric = Chebyshev, weight = distance	0.54	0.54	16	20	29	19

* *Features comprising socio-demographic features, language features, and pronoun features. ^$^top 10 features*.

*ANN, artificial neural network; SVM, support vector machine; kNN, k-nearest neighbors algorithm; TP, true positive; FP, false positive; TN, true negative; FN, false negative*.

**Table 3:** Binary-classification performance for social and emotional support at median and 75 percentile (Leave one out)^*^.

**Target**	**Cutoff**	**AUC**	**F1 Score**	**Top model**	Number of features included*	**Confusion matrix**			
						**TP**	**FP**	**TN**	**FN**
A:Binary-classification performance for social and emotional support at median (Leave one out)^*^.									
Emotional support	3.0	0.72	0.67	ANN ReLu	60	44	18	19	12
Instrumental support	2.0	0.71	0.66	ANN tanh	20	42	18	20	13
Negative social interactions	0.5	0.62	0.67	Tree	30	51	17	12	13
B:Binary-classification performance for social and emotional support at 75 percentiles (Leave one out)^*^.									
Emotional support	3.0	0.72	0.67	ANN ReLu	60	44	18	19	12
Instrumental support	2.5	0.63	0.63	ANN ReLu	5	15	15	44	19
Negative social interactions	1.0	0.63	0.71	SVM	15	20	8	47	18

* *Features comprising socio-demographic features, linguistic features, and pronoun features*.

*TP, true positive; FP, false positive; TN, true negative; FN, false negative*.

We used GINI to rank features for the classification task, the top 10 features results are shown in Table 4. Description of specific features is categorized and grouped in Supplementary Table 3. Several of the top-ranked features were consistently related to loneliness and social support. Lower usage of first-person plural pronouns was linked to higher loneliness, while higher usage of pronouns in general was associated with better emotional and instrumental support, as well as with fewer negative social interactions. Similarly, greater sentence similarity was associated with lower instrumental support while lower sentence complexity was associated with higher loneliness and lower emotional support. Shorter response length in the relationship section was associated with higher loneliness, while shorter responses throughout the interview were associated with lower emotional and instrumental support. Higher education levels were linked to greater loneliness. Lower positive sentiment and higher negative sentiment were consistently linked to less emotional support, less instrumental support, and more negative social interactions.

**Table 4:** Top GINI-ranked predictors in machine learning models for loneliness and social isolation^*^#.

**Loneliness**	**Emotional support**	**Instrumental support**	**Negative social interactions**
First-person plural pronoun (We) (Density, entire transcript)	Response length (Word minimum response)	Sentence similarity (frequency)	Noun usage (frequency)
– *	+	–	–
Compound (positive and negative) sentiment (SD)	Positive sentiment (mean)	Pronoun usage (frequency)	Negative sentiment (SD)
– *	+ ^*^	+	+
Interjection (ratio)	Pronoun usage (Ratio of pronoun to noun)	Negative sentiment (maximum)	Negative sentiment (mean)
–	+ ^*^	+	+ ^*^
Sentence complexity (average yngve depth, Median)	Positive sentiment (median)	Response length (Total number words)	Verb usage (frequency)
– *	+ ^*^	+	–
Response length (Total words, mean)	Compound sentiment (median)	Neutral sentiment (median)	Sentence similarity (median)
–*		–	–
Sentence complexity (yngve depth, Total)	Sentiment neutral (median)	Sentence similarity	Pronoun usage (frequency)
– *	– *	–	–
Response length (Total characters, median)	Sentence complexity (Average yngve depth, median)	Frequency of adjectives	Ratio of nouns
– *	+	+	+
Education	Gender (female)	Response length (Total number characters)	Filler frequency
+ ^*^	+ ^*^	+	–
Total words (Relationship section)	Pronoun usage (ratio)	Verb usage (frequency)	Number of important relationships
–	+ ^*^	+	–
Adverb usage (frequency)	Compound sentiment (mean)	Vocabulary Brunett index	Positive sentiment (SD)
– *	+	+	+

*^$^Features comprising socio-demographic features, language features, and pronoun features*.

* *Significant*.

# *Description of linguistic features is mentioned in Supplementary Appendix A and Supplementary Table 3*.

+ *Associated with higher scores on loneliness, emotional support etc*.

– *Associated with lower scores on loneliness, emotional support, etc*.

*SD, standard deviation*.

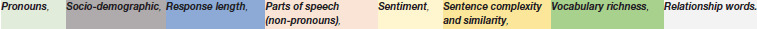

Feature rankings suggest greater role of age than gender in SI/L, with relative information gains of 0.02 vs. 0.01.

## Discussion

Our study explored how text features were associated with SI/L in older community-dwelling adults. Older women's responses to questions about important relationships were more strongly correlated with their ratings on social support scales than older men's. Pronoun density was associated with loneliness and social support in both men and women and were consistently a top feature in models of loneliness and social support. Other top linguistic features included sentence similarity/complexity, response length, and sentiment.

The current finding that usage of first-person plural pronouns was linked to lower loneliness among the women and higher social support among men is consistent with previous research on first-person plural pronoun use as a linguistic indicator of interdependence that has been consistently associated with higher quality relationships and better physical and mental health functioning (33). Studies have also described the links between first-person plural pronoun usage and better perceived support, an expanded sense of self (34), and better conflict resolution in couples (35–38). First-person plural pronoun use also reflects social support within couples, exhibited in how dyads cope together with challenges such as a cancer diagnosis (39–42). Conversely, usage of first-person singular pronoun has been linked with depressive symptoms (43) and negative affective states, noting these associations to be stronger in women (44–46). Language may also influence mood states. Subjects who recalled a depressing incident from a self-distanced perspective (using fewer first-person pronouns) had less depressed affect for up to a week, compared to those who used a more self-immersive stance (47).

The current study illustrates how diverse sets of linguistic features can be used to predict SI/L with good accuracy. The linguistic models presented here (which included a broader variety of linguistic features and sociodemographic information) slightly outperformed our previous models (13), which were limited to NLU-based emotions, sentiment, and question stems from the structured interview template. The current models found that in addition to sentiment, sentence complexity and similarity, usage of pronouns and other parts of speech, and response length were top-ranked features in predicting SI/L. This suggests that a broader variety of linguistic features may outperform purely emotion and sentiment-based models, though more comprehensive models should also include auditory features (e.g., tone, response latency), semantic features (word usage), and longitudinal follow-up. A 2017 study by Mehl et al. (48) reported that lonely individuals used fewer propositions and less time spent talking with others. One study reported that linguistic features such as tentativeness and non-fluencies are associated with depression and anxiety symptoms (49), while another study of Twitter messages found that posts that used “lonely” or “alone” had consistent themes of anger, anxiety, difficulties in interpersonal relationships, substance use, unhealthy eating and sleep (50). One novel study of professional football players and their coaches found longitudinal decline in language complexity in the players (who were at high risk for head trauma) relative to their coaches (51). NLP approaches can capture the breadth of information conveyed through language, augmenting our ability to assess an individual's internal emotional state and social functioning.

All participants were assessed on a wide range of socio-demographic and psychological factors including depression. Previously published studies have shown the overlapping prevalence of depression and loneliness, however due to the low prevalence of depression in this cohort (7.2% had a PHQ-9 score of 10 or greater, 2.1% had a PHQ-9 score of 15 or greater and 0.0% had 20 or greater) and due to a lack of depressive symptoms beyond the mild level of severity, we did not include depression as a confound. For the purposes of this study, only socio-demographic factors and linguistic features were used to predict loneliness and social support.

Our findings included a sizeable number of Facebook users in this age group; it is not very surprising given previous studies that have found older adults to be capable users of technology (52) and, increasingly, social media users—with Facebook use reaching 50% (53) even as younger adults ceased using the social media platform (54).

Several studies have attempted novel techniques to remedy the lack of interpretability of ML models (or their black-box nature). A recent review on the topic, which details the advantages and major drawbacks can be found here (55). Many of these methods have short histories, or, are not widely and openly accepted and/or understood. This is in contrast to ANN models, which are often not only powerful, but they also have a long history, are well and widely understood, studied, and are relatable by most in the field. Most professionals can find a common ground in ANN.

### Study Limitations

Properties of speech (e.g., pitch, prosody, meaningless sounds, amplitude, and modulations) are meaningful features with clinically relevant implications, however, in the current study, we did not assess speech acoustics and relied solely upon the transcribed text.

Our study was cross-sectional and limited to a small sample of independent-living older adults and may not be representative of nor generalizable to the broader class of individuals in the same age group. Our statistical analysis showed a significant age difference between the two genders (Cohen's *d* = −0.68, *p* < 0.001) which potentially confounds age and gender. Follow-up analyses examined the confounding effects of age and gender. Machine learning models exploit combining features in complex non-linear ways to predict the target variables; however, they are difficult to interpret. Linguistic features, by definition, are influenced by language proficiency. Thus, NLP features in non-native English speakers may manifest differently (56, 57). In the current study, we did not control for English proficiency. The models were derived from participants who are fluent in English and may not be applicable to other older adult populations. Pronoun usage may depend on variety of factors such as the number of siblings and size of family when growing up, the choice of profession, and involvement in leadership roles (58). The current study did not control for these factors. Mental health status and momentary emotional state of both, the interviewer and interviewee and their interpersonal dynamics, can influence the interview. Due to a large variety of factors that shape conversations, predictions using these approaches are difficult to perfect.

Character and personality play an important role in verbal expression and are worthy of independent investigation, however this is beyond the scope of the present study. Despite promising initial findings, commonly used sentiment analyzers may be susceptible to bias, due to highly variable assessments, large breadth of applications, or specificity to a particular test case (24).

In this analysis, we have trimmed the least important features, stopping when performance of the model is reduced. While this method of selecting features based on information gain or impurity rank, may result in including features that could be inter-correlated, this does not adversely affect the performance or the results in contrast to traditional statistical methods. This method may not provide the minimal feature set, which is very difficult to identify (59), but roughly identified sets such as ours work well in practice.

For this project, we aimed to compare language usage differences between people with and without SI/L. Transformers, despite being very useful in certain cases that require extraction of meaning, have limited applicability in our study. First, they are intended to process text, not linguistic features. Second, transformers are uniquely equipped for tasks such as translation and summarizing as they are designed to retain meaningful concepts using attention (60). But this has an effect of deemphasizing less important details, which have less to do with the meaning but more to do with expression e.g., vocabulary richness, filler words, and pronouns. Third, recent studies have reported that Bidirectional Encoder Representations from Transformers (BERT—a well-known architecture that first introduced the idea of attention and was quickly embraced by the community) often cannot outperform some common classification and other simpler baselines (61–63). Crafting an appropriate transformer for the task may not be straightforward, and advantages may translate into just a few percentage points in performance.

### Future Directions/Overall Conclusions

The application of NLP for the purpose of facilitating understanding of human health is exciting. The fact that myriad factors can influence conversations, more research is needed to refine the predictive accuracy of these models. NLP assessments of unstructured language may be integrated with self-report and behavioral assessments to provide nuanced and sensitive evaluations of SI/L. Moreover, the narrative data that forms the basis of the NLP training data must be evaluated to ensure that it is representative of people for whom the results may be applied. Given its novelty, those exploring NLP applications, including researchers and clinicians, should become knowledgeable about how to approach its use and consider issues of bias, fairness, accountability, and related ethical and social implications early and often during the study. While this study was limited to common architectures used in ML, newer attention-based models, such as transformers, may provide additional improvements.

Due to low rates of depression in this cohort, we were not able to assess language features that were reflective of depressive symptoms. However, future NLP studies of lonely cohorts with higher rates of depression should consider how the impact of depression on language, both independent of SI/L as well as through effects on social functioning.

## Data Availability Statement

The study/data is governed by University of California San Diego Human Research Protections Program (HRPP) rules and other contract. It is not publicly available due to privacy concerns, may include HIPAA regulations. For access, qualified researchers may contact the corresponding author.

## Ethics Statement

The studies involving human participants were reviewed and approved by University of California San Diego Human Research Protections Program. The patients/participants provided their written informed consent to participate in this study.

## Author Contributions

VB and EL contributed to the conception and design of the study and had full access to all the data in the study and take responsibility for the integrity of the data and the accuracy of the data analysis. VB wrote the first draft of the manuscript and conducted the data analyses. VB, YY, and KS developed the NLP tools used in the analyses. EL, VB, CN, YY, KS, KR, and H-CK were involved in the data interpretation. VB, EL, and CN wrote sections of the manuscript and were involved in data interpretation. All authors contributed to manuscript revision, read, and approved the submitted version.

## Funding

This study received funding from IBM. The funder was not involved in the study design, collection, analysis, interpretation of data, the writing of this article or the decision to submit it for publication.

## Conflict of Interest

KS, YY, and H-CK are employees of IBM. The remaining authors declare that the research was conducted in the absence of any commercial or financial relationships that could be construed as a potential conflict of interest.

## Publisher's Note

All claims expressed in this article are solely those of the authors and do not necessarily represent those of their affiliated organizations, or those of the publisher, the editors and the reviewers. Any product that may be evaluated in this article, or claim that may be made by its manufacturer, is not guaranteed or endorsed by the publisher.
