# Supplementary material for: Do Words Matter? Detecting Social Isolation and Loneliness in Older Adults Using Natural Language Processing
Source: Front Psychiatry. 2021 Nov 16;12:728732. doi: 10.3389/fpsyt.2021.728732 (PMC8635064; doi:10.3389/fpsyt.2021.728732)
Supplement: Supplementary file 1 [file Data_Sheet_1.docx]

**Supplementary Materials**

**Supplemental Appendix A: Description of Linguistic Features**

**Preprocessing**

For preprocessing, we performed sentence tokenization, word tokenization, and part-of-speech tagging on the transcribed text by using the Natural Language Toolkit (NLTK) [7] modules integrated with Python. We also performed lemmatization using Wordnet lemmatizer [23] available on NLTK.

**Linguistic features**

- Vocabulary Richness

The features related to vocabulary richness measure lexical diversity. They were ported to decline in patients with Alzheimer’s disease [1, 2] and mild cognitive impairment [3].

There are three types of vocabulary-richness features: type-token ratio (TTR), Brunét’s index (BI), and Honoré’s statistic (HS) [4]. TTR compares the total distinct word types (U) to the total word count (V) as $\mathrm{TTR}=U/V$. Using the same U and V, BI is defined as $\mathrm{BI}=V^{U^{-0.165}}$. Unlike other measures related to vocabulary richness, for this measure, the lexical richness becomes greater as BI becomes smaller. HS gives particular importance to unique vocabulary items used only once, also known as hapax legomena $V_{uni}$. HS is defined as $\mathrm{HS}=100logV/(1-V_{uni}/U)$.

- Part-of-speech

Frequency and proportion of part-of-speech has been used as linguistic features. For example, pronoun frequency has been used for quantifying difficulties with word-finding and word-retrieving in patients with Alzheimer’s disease and mild cognitive impairment. Previous studies on Alzheimer’s patients’ speech reported increased proportion of pronouns [1,4-6], adjective [4, 6] and verbs [4, 6] and reduced proportion of nouns [4, 6]. We used frequency and proportion of the following part-of-speech tags as linguistic features: nouns, verbs, adjectives, pronouns, adverbs, conjunctions, particles, and interjections. About the proportion, we normalized the frequency by the total number of words. In addition, we used as features the proportion of nouns to verbs, pronouns to nouns, nouns to adjectives, and verbs to adjectives.

Pronoun usage is especially informative on how an individual views himself or herself in relationship to others. We specifically examined the proportion of first-person singular pronouns (I, me, my, mine), first-person plural pronoun (we, our, us, ours) and the third-person pronouns (he, she, they, them, their) to investigate differences in the two groups. We excluded second person pronouns (you, your, yours) as second person pronouns were primarily reserved to address the interviewer (or vice-versa) in these transcripts. We calculated the counts and frequencies of such usage initially adhering to the relationship section, as we had done for other features, but found out that the values were not very dissimilar. When we studied the usages across the entire transcript, the usages were meaningfully different. We attributed this to the section’s unusual focus on people.

- Filled pause

Frequency and proportion of filled pauses have been used for measuring dysfluency in speech and quantifying language dysfunctions related to impaired lexical access, syntactic difficulties, discourse planning deficits [8]. They were reported to increase in case of Alzheimer’s disease [9], Aphasia [10] and depression [11].

- Syntactic complexity

Features related to syntactic complexity have been used for quantifying syntactic impairments as well as memory and semantic difficulties [1, 12]. Several studies have found a decrease in the syntactic complexity of language in case of Alzheimer’s disease [2, 13, 14] and Aphasia [10]. Features related to syntactic complexity include length metrics and parse tree metrics. As for the length metrics, we used total number of sentences, words, and characters as well as their statistics across sentences including mean, median, minimum, maximum, standard deviation. Parse tree metrics include statistics of the Yngve depth (a measure of embeddedness) across sentences [15] and its total depth. Yngve depth was calculated by using Stanford CoreNLP package [16].

- Sentence similarity

Sentence similarity has been used for quantifying repetition or perseveration in the speech. Previous studies on patients with Alzheimer’s disease reported increased sentence similarities associated with frequent repetition of specific words and phrases [17, 18].

One of the sentence similarity measures is calculated by using cosine similarity between sentences. We first converted sentences into term frequency–inverse document frequency (TF-IDF) vectors by using a bag-of-words model [19]. TF-IDF is a numerical statistic intended to reflect how important a word is to a document. By using TF-IDF vectors, we then calculated the cosine similarity between all pairs of sentences and used their statistics as well as the frequency and proportion of similar sentence pairs (>0.5). The statistics included mean, median, minimum, maximum, and standard deviation.

- Sentiment

Sentiment features typically used for detecting changes in mental states. For example, depressed individuals were reported to tend to use more negative words and fewer positive words than non-depressed individuals [20, 21]. We used statistics of positive, negative, and neutral emotional scores for each sentence as well as a compound score for the document. The compound score ranges from -1 (most negative) to +1 (most positive). The scores were calculated by using VADER sentiment analyzer [22] with Python NLTK package [7].

**References:**

[1] Fraser, Kathleen C., Jed A. Meltzer, and Frank Rudzicz. "Linguistic features identify Alzheimer’s disease in narrative speech." Journal of Alzheimer's Disease 49.2 (2016): 407-422.

[2] Yancheva, Maria, Kathleen C. Fraser, and Frank Rudzicz. "Using linguistic features longitudinally to predict clinical scores for Alzheimer’s disease and related dementias." Proceedings of SLPAT 2015: 6th Workshop on Speech and Language Processing for Assistive Technologies. 2015.

[3] Beltrami, Daniela, et al. "Speech analysis by natural language processing techniques: a possible tool for very early detection of cognitive decline?." Frontiers in aging neuroscience 10 (2018): 369.

[4] Bucks RS, Singh S, Cuerden JM, Wilcock GK. Analysis of spontaneous, conversational speech in dementia of Alzheimer type: evaluation of an objective technique for analysing lexical performance. Aphasiology 2000 Jan;14(1):71-91.

[5] Ahmed, Samrah, et al. "Connected speech as a marker of disease progression in autopsy-proven Alzheimer’s disease." Brain 136.12 (2013): 3727-3737.

[6] Jarrold, William, et al. "Aided diagnosis of dementia type through computer-based analysis of spontaneous speech." Proceedings of the Workshop on Computational Linguistics and Clinical Psychology: From Linguistic Signal to Clinical Reality. 2014.

[7] Bird, Steven. "NLTK: the natural language toolkit." Proceedings of the COLING/ACL 2006 Interactive Presentation Sessions. 2006.

[8] Boschi, Veronica, et al. "Connected speech in neurodegenerative language disorders: a review." Frontiers in psychology 8 (2017): 269.

[9] Khodabakhsh, Ali, et al. "Evaluation of linguistic and prosodic features for detection of Alzheimer’s disease in Turkish conversational speech." EURASIP Journal on Audio, Speech, and Music Processing 2015.1 (2015): 1-15.

[10] Ash, Sharon, et al. "Differentiating primary progressive aphasias in a brief sample of connected speech." Neurology 81.4 (2013): 329-336.

[11] Stasak, Brian. "An Investigation of Acoustic, Linguistic, and Affect Based Methods for Speech Depression Assessment." (2018).

[12] Reilly, Jamie, Joshua Troche, and Murray Grossman. "Language processing in dementia." The handbook of Alzheimer’s disease and other dementias 7 (2011).

[13] Orimaye, Sylvester Olubolu, Jojo Sze-Meng Wong, and Karen Jennifer Golden. "Learning predictive linguistic features for Alzheimer’s disease and related dementias using verbal utterances." Proceedings of the workshop on computational linguistics and clinical psychology: From linguistic signal to clinical reality. 2014.

[14] Pakhomov, Serguei, et al. "Computerized assessment of syntactic complexity in Alzheimer’s disease: A case study of Iris Murdoch’s writing." Behavior research methods 43.1 (2011): 136-144.

[15] Yngve, Victor H. "A model and an hypothesis for language structure." Proceedings of the American philosophical society 104.5 (1960): 444-466.

[16] Manning, Christopher D., et al. "The Stanford CoreNLP natural language processing toolkit." Proceedings of 52nd annual meeting of the association for computational linguistics: system demonstrations. 2014.

[17] Nicholas, Marjorie, et al. "Empty speech in Alzheimer's disease and fluent aphasia." Journal of Speech, Language, and Hearing Research 28.3 (1985): 405-410.

[18] Tomoeda, Cheryl K., et al. "Cross-sectional analysis of Alzheimer disease effects on oral discourse in a picture description task." Alzheimer disease and associated disorders (1996).

[19] Masrani, Vaden, et al. "Detecting dementia through retrospective analysis of routine blog posts by bloggers with dementia." BioNLP 2017. 2017.

[20] Scibelli, Filomena. "Detection of Verbal and Nonverbal speech features as markers of Depression: results of manual analysis and automatic classification." (2019).

[21] Morales, Michelle, Stefan Scherer, and Rivka Levitan. "A cross-modal review of indicators for depression detection systems." Proceedings of the fourth workshop on computational linguistics and clinical psychology—From linguistic signal to clinical reality. 2017.

[22] Hutto, Clayton, and Eric Gilbert. "Vader: A parsimonious rule-based model for sentiment analysis of social media text." Proceedings of the International AAAI Conference on Web and Social Media. Vol. 8. No. 1. 2014

[23] George A. "WordNet: a lexical database for English." Communications of the ACM 38.11 (1995): 39-41.

**Supplemental Appendix B: A note on TF-IDF and representing documents as vectors**

A piece of text can be represented as a vector (a sequence of numbers, possibly long, but finite), in which each position represents a word in the dictionary (by position) and contains the count of that word in the text, or zero if it does not. This count is called “frequency” or Term Frequency (TF). Not all words are used with equal frequency. We divided each entry in the TF vector by the number of documents in which it appears or Document Frequency (DF), to assign greater importance to rarely used words. See (Scott, 2019) for some informal details

**Supplemental Table 1: Relationship dictionary**

| **Dictionary^*^** | **Category** |
| --- | --- |
| ‘friend’ | Friend |
| 'daughter', 'son', 'child', 'stepson', 'stepdaughter', 'stepchild', 'kid', 'goddaughter', 'godson', 'girl', 'boy' | Child |
| 'grandbaby', 'granddaughter', 'grandson', 'grandchild', 'grandkid' | Grandchild |
| 'great-granddaughter', 'great-grandson', 'great-grandchild', 'great-grandkid' | Great-grandchild |
| 'husband', 'wife', 'spouse' | Spouse |
| 'sibling', 'sister', 'brother', 'stepsister', 'stepbrother' | Sibling |
| 'family' | Family |
| 'relative', 'niece', 'nephew', ‘great-niece’, ‘great-nephew’, 'cousin', 'aunt', 'uncle' | Relative |
| 'parent', 'mom', 'dad', 'mother', 'father' | Parent |
| 'jesus', 'god', 'church-community', | Spiritual |
| 'girlfriend', 'boyfriend', 'partner' | Partner |
| 'daughter-in-law', 'son-in-law', 'sister-in-law', 'brother-in-law', 'mother-in-law', 'father-in-law' | In-laws |
| 'dog', ‘cat’ | Pet |
| 'roommate', 'resident', 'assistant', ‘teacher’, ‘mentor’, ‘professor’ | Other |

**^*^**Only singular form is mentioned here

**Supplemental Table 2: Mapping of responses into equivalent weekly frequency.**

| **Response** | **Weekly frequency** | **Monthly frequency** | **Category** |
| --- | --- | --- | --- |
| constantly | 14 | 60 | Very frequently |
| all the time | 14 | 60 | Very frequently |
| awful lot | 14 | 60 | Very frequently |
| lot of time | 14 | 60 | Very frequently |
| daily | 7 | 30 | Frequently |
| everyday | 7 | 30 | Frequently |
| most days | 5 | 21.4 | Frequently |
| times a week | 2 | 8.6 | Moderately |
| weekly | 1 | 4.3 | Moderately |
| once a week | 1 | 4.3 | Moderately |
| once in a while | 0.5 | 2.1 | Moderately |
| a month | 0.25 | 1.1 | Moderately |
| months | 0.2 | 0.9 | Infrequently |
| couple of months | 0.2 | 0.9 | Infrequently |
| not a whole lot | 0.1 | 0.4 | Infrequently |
| not a lot | 0.1 | 0.4 | Infrequently |
| very little | 0.1 | 0.4 | Infrequently |

**Supplemental Table 3: Description, sources, and interpretation of top NLP features**

| **Feature Name** | **Feature Type*** | **Our Interpretation** | **References for computation of features** |
| --- | --- | --- | --- |
| **Pronoun Features** | | | |
| “We” pronoun usage (density) | Part-of-speech | Self in relation to others |  |
| Third-person pronoun | Part-of-speech | Self in relation to others, quantity of relationships |  |
| Pronoun usage (ratio of pronoun to noun) | Part-of-speech | Number of people referred to in conversation | 1 |
| Pronoun usage (ratio) | Part-of-speech |  | 1 |
| Pronoun usage (frequency) | Part-of-speech |  | 1 |
| **Demographics (education)** | | | |
| Education (total years) | **Other**** |  |  |
| Veteran status | **Other**** |  |  |
| **Response Length** | | | |
| Response length (total number of words) | Syntactic Complexity | Talkativeness, openness, interest in conversation | 1 |
| Response length (minimum) | Syntactic Complexity | Talkativeness, openness, interest in conversation | 1 |
| Response length (median characters) | Syntactic Complexity | Talkativeness, openness, interest in conversation | 1 |
| Response length (total number characters) | Syntactic Complexity | Talkativeness, openness, interest in conversation | 1 |
| **Non pronoun** | | | |
| Ratio of interjections | Part-of-speech |  | 1 |
| Frequency of conjunctions | Part-of-speech | Conveying a list of ideas | 1 |
| Ratio of verbs | Part-of-speech |  | 1 |
| Ratio of adjectives | Part-of-speech | Ability to describe in detail | 1 |
| Ratio of Nouns to Adjectives | Part-of-speech | Ability to describe in detail | 1 |
| Frequency of adjectives | Part-of-speech |  | 1 |
| Frequency of fillers | Part-of-speech |  | 1 |
| Frequency of verbs | Part-of-speech |  | 1 |
| Ratio of nouns | Part-of-speech | memory | 1 |
| Frequency of nouns | Part-of-speech | memory | 1 |
| **Sentiment** | | | |
| Compound sentiment (SD) | VADER*** |  | 2 |
| Positive sentiment (mean) | VADER*** |  | 2 |
| Neutral sentiment (median) | VADER*** |  | 2 |
| Positive sentiment (median) | VADER*** |  | 2 |
| Negative sentiment (maximum) | VADER*** |  | 2 |
| Negative sentiment (mean) | VADER*** |  | 2 |
| Negative sentiment (SD) | VADER*** |  | 2 |
| Positive sentiment (SD) | VADER*** |  | 2 |
| **Semantic similarity** | | | |
| Cosine similarity (0.5 ratio) | **Perseveration** | Not digressing from original idea | 1 |
| Cosine similarity (0.5 freq) | **Perseveration** | Not digressing from original idea | 1 |
| **Semantic complexity** | | | |
| yngve_avg_depth_median | Syntactic Complexity | Ability to construct and convey nested ideas | 1 |
| yngve_depth_total | Syntactic Complexity | Ability to construct and convey nested ideas | 1 |

*Category mentioned in Multimedia Appendix 1 in [1] when available, marked otherwise.

**Sociodemographic information from patient records

***VADER. “Valence Aware Dictionary and sEntiment Reasoner” – A python library for sentiment

Analysis

[1] Description of linguistic features is mentioned in (Yamada, Shinkawa, & Shimmei, 2020) and Appendix A.

[2] (Hutto & Gilbert, 2014)

**References**

Hutto, C., & Gilbert, E. (2014). Vader: A parsimonious rule-based model for sentiment analysis of social media text *Proceedings of the International AAAI Conference on Web and Social Media* (Vol. 8).

Scott, W. (2019). *TF-IDF from scratch in python on real world dataset.* Retrieved from <https://towardsdatascience.com/tf-idf-for-document-ranking-from-scratch-in-python-on-real-world-dataset-796d339a4089>

Yamada, Y., Shinkawa, K., & Shimmei, K. (2020). Atypical Repetition in Daily Conversation on Different Days for Detecting Alzheimer Disease: Evaluation of Phone-Call Data From a Regular Monitoring Service. *JMIR mental health, 7*(1), e16790
